# Hydrogen sulfide attenuates sepsis-induced cardiac dysfunction in infant rats by inhibiting the expression of cold-inducible RNA-binding protein

**DOI:** 10.1042/BSR20241398

**Published:** 2025-02-17

**Authors:** Desi Li, Sheng Jin, Xu Teng, Ping Wang, Kaichuan He, Lijing Cao, Jiexian Du, Qi Guo, Lin Xiao, Hongmei Xue, Danyang Tian, Cuixia An, Yuming Wu

**Affiliations:** 1 Department of Physiology, Hebei Medical University, Hebei 050017, China; 2 Department of Medical, Hebei Medical University Third Hospital, Hebei 050051, China; 3 Clinical Medicine Research Center, Hebei General Hospital, Hebei 050051, China; 4 Department of Pediatric Intensive Care Unit, Hebei Children’s Hospital, Hebei 050031, China; 5 Gynecology and Obstetrics, The Second Hospital of Hebei Medical University, Hebei 050000, China; 6 Department of Psychiatry, The First Hospital of Hebei Medical University, Hebei 050000, China; 7 Hebei Collaborative Innovation Center for Cardio-Cerebrovascular Disease, Hebei 050031, China; 8 The Key Laboratory of Neural and Vascular Biology, Ministry of Education, Hebei 050017, China; 9 Hebei Key Laboratory of Cardiovascular Homeostasis and Aging, Hebei 050017, China

**Keywords:** CLP‐induced cardiac dysfunction, cold-inducible RNA-binding protein, endoplasmic reticulum stress, hydrogen sulfide, pediatric sepsis

## Abstract

Sepsis-induced cardiac dysfunction is one of the most common complications of sepsis. It is also a major cause of death in pediatric intensive care units. The underlying mechanism of sepsis-induced cardiac dysfunction remains elusive. Cold-inducible RNA-binding protein (CIRP) is a damage-associated molecular pattern that is up-regulated during sepsis. Hydrogen sulfide (H_2_S) has been shown to play a protective role in sepsis‐induced cardiac dysfunction in adult animals. The present study aimed to determine whether H_2_S ameliorates the cardiac function in infant rats by inhibiting CIRP-mediated sepsis-induced cardiac dysfunction. Rat pups aged 17–18 days were subjected to cecal ligation and puncture (CLP) to induce sepsis. Six hours after CLP, hemodynamic results demonstrated that there was a significant decrease in +dP/dt_max_, −dP/dt_max_, left ventricular ejection fraction, and left ventricular shortening fraction, indicating cardiac dysfunction. The plasma levels of myocardial injury markers such as creatine kinase–myocardial band and cardiac troponin I were significantly increased at 6 h after CLP. The inhibition of CIRP with C23 improved the cardiac function of the rats with CLP-induced sepsis, accompanied by a significant decrease in endoplasmic reticulum stress (ERS) activation. Moreover, treatment with sodium 4-phenylbutyrate (an inhibitor of ERS) ameliorated myocardial injury and dysfunction, accompanied by a significant decrease in ERS activation. Sodium hydrosulfide, a H_2_S donor, ameliorated CLP-induced cardiac dysfunction and decreased CIRP levels and ERS. In contrast, the inhibition of endogenous H_2_S production by propargylglycine (a cystathionine-γ-lyase inhibitor) aggravated CLP-induced cardiac dysfunction and increased CIRP levels. In conclusion, the present study demonstrated that H_2_S exerted cardioprotective effects by inhibiting the CIRP/ERS pathway in infant rats with sepsis. These findings might indicate a novel target in the treatment of sepsis in infants.

## Introduction

Sepsis is a common medical condition that severely damages the health of children. Severe sepsis is considered a major cause of death from infection in children worldwide [1]. Sepsis is a life-threatening multiple-organ dysfunction caused by dysregulated host immune responses to infection. More than 7000 pediatric deaths due to sepsis occur in the United States each year [2]. A recent report stated that the mortality rates were more than 10% in developing countries (31.7%), which is higher than the mortality rates in developed countries (19.3%) [3]. Sepsis constitutes a considerable health and financial burden globally. Therefore, increasing attention has been focused on sepsis throughout the world. In 2017, the World Health Assembly passed a resolution calling for improvements in the prevention, diagnosis, and management of sepsis.

Sepsis-induced cardiac dysfunction is a common complication of severe sepsis and septic shock. Patients often present with diastolic dysfunction and decreased ejection fraction [4]. In a previous study, sepsis was accompanied by cardiac dysfunction in approximately 40–44% of adult patients and 80% of pediatric patients [5]. Septic patients with cardiac dysfunction have significantly higher mortality rates than patients without this condition [6]. The presence of cardiac dysfunction in sepsis is associated with a 20–50% increase in mortality rate in patients with sepsis [7]. Therefore, improved cardiac function plays an essential role in reducing mortality in pediatric patients with sepsis. Although considerable effort has been made to identify biochemical mediators or a pathway, the specific cause of myocardial depression in sepsis remains unclear.

Increasing evidence has shown that sepsis is a complex syndrome caused by an intense immune response to infection [8]. This complex process is triggered by exogenous pathogen-associated molecular patterns (PAMPs) or endogenous damage-associated molecular patterns (DAMPs) [9]. Innate immunity initiated via PAMPs or DAMPs is recognized by pathogen recognition receptors, which are expressed on innate immune cells [10]. Previous studies have indicated that DAMPs are associated with the severity of clinical sepsis [11,12]. Cold-inducible RNA-binding protein (CIRP) has been described as an RNA chaperone that facilitates translation is response to some stressful conditions, such as mild hypothermia, hypoxia, and exposure to ultraviolet irradiation [13]. A recent study demonstrated that CIRP could promote inflammatory responses in hemorrhagic shock and sepsis. CIRP could act as a DAMP [14]. CIRP could bind to the TLR4/MD2 receptor complex and activates the TLR4/MyD88/NF-κB pathway, leading to increased severity and mortality rates. However, whether and how CIRP participates in sepsis-induced cardiac dysfunction in children remains unclear.

The endoplasmic reticulum (ER) is a crucial cellular organelle. It plays vital roles in maintaining intracellular calcium homeostasis, protein metabolism, and post-translational modifications [15]. Perturbations in ER homeostasis by various physiological stimuli (infections, calcium dysregulation, ischemia, and hypoxia) lead to the accumulation of misfolded proteins. This pathological process is referred to as ER stress (ERS) [16]. ERS is also associated with the inflammatory process. Research has shown that inhibiting ERS could improve the overall survival of patients with sepsis [17]. The mechanism by which CIRP participates in stress is related to ERS [18]. In sepsis-associated acute lung injury, ERS was induced by CIRP [19]. However, how CIRP affects ERS in infant sepsis-induced cardiac dysfunction remains unknown.

The gasotransmitter hydrogen sulfide (H_2_S) is a signaling molecule similar to nitric oxide (NO) and carbon monoxide (CO) [20]. Endogenous H_2_S in mammals is mostly generated by cystathionine γ-lyase (CSE), cystathionine β-synthase, and 3-mercaptopyruvate sulfurtransferase [13]. H_2_S plays an important role in regulating the cardiovascular system. Accumulating evidence has demonstrated the anti-inflammatory activity of H_2_S. In our previous study, we showed that H_2_S protected kidney function by alleviating oxidative stress and attenuating inflammation in sepsis-induced acute kidney injury [21]. However, there is a paucity of studies on the role of H_2_S in children with sepsis.

In the present study, we demonstrated that H_2_S attenuates inflammation, ERS, and histological injury in the hearts of infant rats. Overall, these findings suggest that H_2_S plays an active role by inhibiting CIRP and ERS expression in sepsis-induced cardiac dysfunction in infant rats.

## Materials and methods

### Animals

All animal procedures were approved by the Animal Management Rule of the Ministry of Health, People’s Republic of China (documentation number 55, 2001) and the Animal Care Committee of Hebei Medical University. The protocols and procedures performed were in compliance with the Guide for the Care and Use of Laboratory Animals (NIH Publication No. 85-23, revised 2011).

The present study was conducted at Hebei Medical University. All studies were performed with 17–18-day-old male rats (37–43 g). All animals were obtained from the Animal Center of Hebei Medical University (Shijiazhuang, China). The animals were housed under standard conditions (room temperature, 20–25°C; humidity, 50–65%; and a 12-h light/dark cycle) and were freely supplied with standard chow and water. The present study was conducted in accordance with the Helsinki Declaration and was approved by the Ethics Committee of Hebei Medical University Third Hospital, No. W2021-056-1.

### Animal model of sepsis

Severe sepsis was induced by cecal ligation and puncture (CLP), which was performed as previously described [22]. The animals were randomly categorized into either a CLP group or a sham group. They were anesthetized with 1% isoflurane, after which laparotomies were performed to expose their ceca. A 4-0 silk suture was used to ligate 1.5 cm of the cecal tip, ensuring not to ligate the ileocecal valve so as to not cause bowel obstruction. The cecum was punctured through-and-through twice with an 18 G needle. Approximately 1 mm of fecal material was released from each perforation. Sham group rats underwent middle laparotomy but with no CLP. The abdominal incision was closed in all animals, and 25 ml/kg warm saline (i.p.) was administered postoperatively. The rats were placed in separate cages and were then further randomized to receive vehicle (saline), 100 mg/kg 4-PBA (MCE, NJ, U.S.A.), 8 mg/kg C23 (GRGFSRGGGDRGYGG; synthesized from GL Biochem, Shanghai, China), 25 μmol/kg NaHS (Sigma-Aldrich, St Louis, MO, U.S.A.), or 25 mg/kg PPG (Sigma-Aldrich) 1 h after surgery. At 6-h interval after CLP, the core body temperature was measured using a rectal thermometer; then, measurement of blood pressure and hemodynamic parameters were performed as described below, after which cardiac tissues were harvested.

### Measurements of blood pressure and cardiac function

Six hours postoperatively, the rats were anesthetized using 1% isoflurane and conducted to carotid catheterization. A pressure transducer (ML4818) connected to a power lab apparatus (AD Instruments, Australia) was used for measuring blood pressure, heart rate, and cardiac function. A polyethylene catheter was introduced from the right carotid artery, and blood pressure was recorded for 5–10 min. Then, a catheter (retrograde) was introduced through the aortic valve to reach the left ventricular (LV) cavity, after which LV pressure was recorded for 15–30 min. All data were recorded and analyzed using Lab Chart 7 software. The rate of increase in LV pressure during systole (+dP/dt_max_) and the maximal negative slope at the diastole phase (−dP/dt_max_) were acquired from the pressure waves. At the experimental endpoint, mice were anesthetized using 1% isoflurane and subsequently sacrificed during anesthesia by intraperitoneal injection of a lethal dose of thiopental (200 mg/kg) followed by cervical dislocation, and blood and heart tissues were collected and stored.

### Echocardiographic assessment

The echocardiogram was performed using a Vevo 2100 ultrasound device (FUJIFILM Visual Sonics Inc., Toronto, Canada). The rats were anesthetized with 1% isofluran. Parasternal long and short axis images were acquired in both two-dimensional and M-mode for quantitative analysis, while LV ejection fraction (LVEF) and LV shortening fraction (LVFS) were calculated through computer algorithms. Measurements over three consecutive cardiac cycles were averaged.

### Measurement of plasma CK-MB levels

Plasma creatine kinase–myocardial band (CK-MB) levels in rats were determined using an Assay Kit (Jiancheng, NanJing, China).

### Measurement of plasma cTnI levels

Enzyme-linked immunosorbent assay kits were used to determine plasma cTnI (Elabscience, Wuhan, China) levels in rats. All procedures were performed according to the manufacturer’s instructions.

### Morphological studies

After hemodynamic parameters were measured, the thorax was opened rapidly and the heart was removed and washed with ice-cold saline. Briefly, rat heart tissues were fixed in 4% paraformaldehyde solution and embedded in paraffin; then, tissue samples were cut into 5-µm sections and stained with hematoxylin and eosin (H&E). Finally, the morphology of the sections was observed by a blinded observer using a light microscope (DM6000B, Leica Microsystems). The sections were evaluated by a blinded pathologist to assess the severity of the injury.

### Western blotting

Frozen heart tissues were lysed mechanically in cold RIPA buffer. Proteins were extracted and quantified using a BCA protein assay kit (BestBio, Shanghai, China). The proteins were electrophoretically separated using SDS-PAGE and transferred to a polyvinylidene difluoride membrane (Millipore, U.S.A.). The membranes were blocked with 3% BSA for 1.5 h at room temperature, and antigens were detected using the following antibodies at 4°C overnight: GRP78 (1:10000, Santa Cruz Biotechnology Inc., TX, U.S.A., sc-166490), ATF6 (1:500, MCE, SH, China, HY-P80379), p-PERK (1:1000, GeneTex, SZ, China, GTX00673), PERK (1:1000, Santa Cruz Biotechnology, TX, U.S.A., sc-377400), p-IRE1-a (1:1000, Abcam; Cambridge, MA, U.S.A., ab48187), IRE1-a (1:1000, Abcam; Cambridge, MA, U.S.A., ab37073), CIRP (1:5000, Proteintech Biotechnology, WH, China, 10209-2-AP), CSE (1:1000, Proteintech Biotechnology, WH, China, 60234-1-Ig), and GAPDH (1:5000, Proteintech Biotechnology, WH, China, 10494-1-AP). The blots were incubated with horseradish peroxidase-conjugated anti-rabbit (1:5000, Proteintech Biotechnology, WH, China, SA00001-2) or anti-mouse (1:5000, Proteintech Biotechnology, WH, China, SA00001-1) secondary antibody at room temperature for 1.5 h. All antibody dilutions were performed using TBST. Blot bands were visualized using ECL detection system and detected using ImageJ software (ImageJ 1.52, NIH, U.S.A.).

### Statistical analyses

Statistical analysis was performed using SPSS 21 software (SPSS Inc., Chicago, IL, U.S.A.). Data were presented as mean ± SD, all experiments include six biological replicates (*n* = 6), and independent *t*-tests were used to perform comparisons between two groups. The results of more than three groups were compared using one-way analysis of variance followed by LSD (Least-Significant Difference) test with equal variance, and the Dunnett’s T3 method was performed if there was missing variance. *P*-values of <0.05 were considered statistically significant.

## Results

### CLP-induced sepsis caused myocardial dysfunction in the infant rats

**Figure 1:**
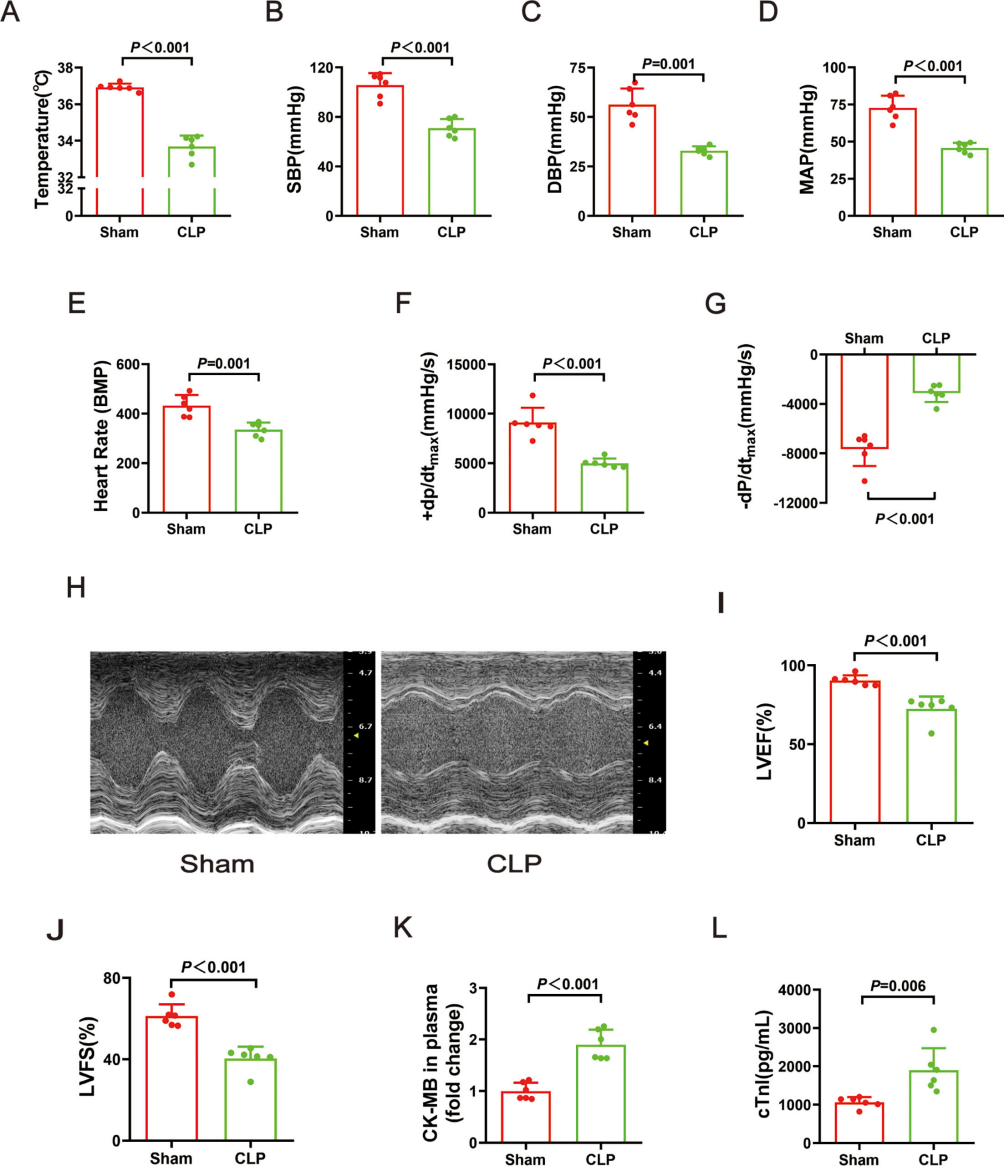
Cecal ligation and puncture induced cardiac function changes in infant rats. (**A**) The change of temperature. (**B**) The change of SBP. (**C**) The change of DBP. (**D**) The change of MAP. (**E**) The change of heart rate. (**F**) The change of +dP/dt_max_. (**G**) The change of -−dP/dt_max_. (**H**) Representative images of M-mode by echocardiography. (**I**) The change of LVEF. (**J**) The change of LVFS. (**K**) The fold change of CK-MB in plasma. (**L**) The level of cTnI in plasma. Data are mean ± SD from two independent experiments (*n* = 6). Independent *t*-tests, *P*＜0.05 was considered significant vs. Ssham. CK-MB, creatine kinase–myocardial band; DBP, diastolic blood pressure ; LVEF, left ventricular ejection fraction; LVFS, left ventricular fractional shortening; MAP, mean arterial blood pressure; SBP, systolic blood pressure.

The rats in the CLP group had significantly lower core temperatures, systolic blood pressure (SBP), diastolic blood pressure (DBP), and mean arterial blood pressure (MAP) than those in the sham group (Figure 1A–D). There was also a marked reduction in the heart rate (Figure 1E). The LV function was assessed by analyzing LV pressure waves obtained through a catheter that was inserted into the right carotid artery and advanced into the LV, in conjunction with echocardiography. Maximum contraction velocity (+dP/dt_max_), maximum relaxation velocity (−dP/dt_max_), LVEF, and LVFS were significantly decreased in the CLP group relative to those in the sham group (Figure 1F–J). Furthermore, plasma CK-MB and cTnI levels were significantly higher in the CLP group than in the sham group (Figure 1K–L). These results demonstrate that CLP-induced sepsis in infant rats caused myocardial dysfunction.

### Inhibition of CIRP expression could reduce sepsis-induced cardiac dysfunction and ERS

**Figure 2:**
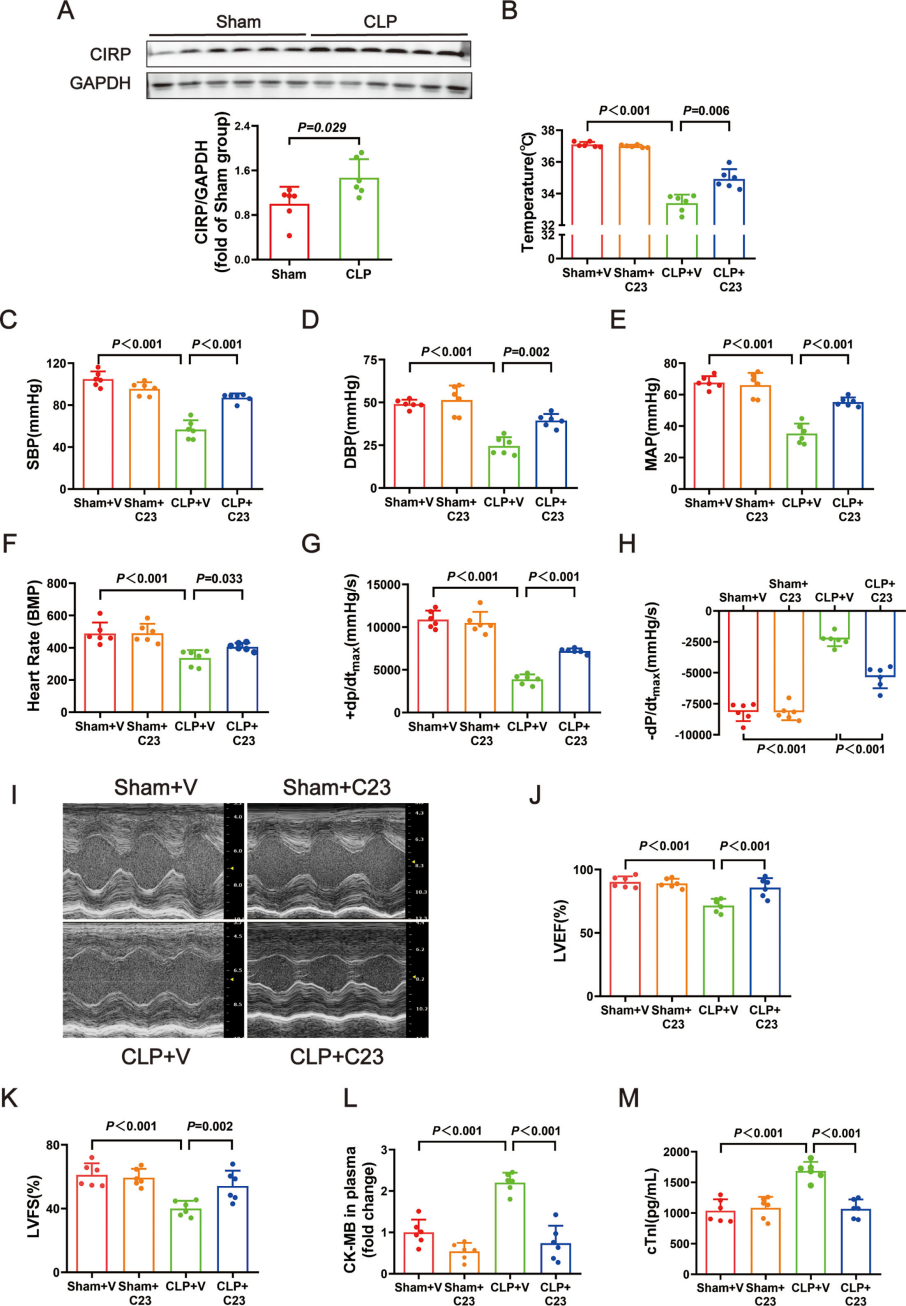
Treatment with C23, an inhibitor of CIRP, improved cardiac function in septic infant rats. (**A**) Representative Western blots and quantitative analysis for CIRP. GAPDH was used as the internal control. (**B**) The change of temperature. (**C**) The change of SBP. (**D**) The change of DBP. (**E**) The change of MAP. (**F**) The change of heart rate. (**G**) The change of +dP/dt_max_. (**H**) The change of -−dP/dt_max_. (**I**) Representative images of M-mode by echocardiography. (**J**) The change of LVEF. (**K**) The change of LVFS. (**L**) The fold change of CK-MB in plasma. (**M**) The level of cTnI in plasma. Data are mean ± SD from two independent experiments (*n* = 6). Independent *t*-tests, *P*＜0.05 was considered significant vs. Ssham. Data are mean ± SD from four independent experiments (*n* = 6). One-way analysis of variance followed by LSD test with equal variance, and the Dunnett’s T3 method was performed if there was missing variance, *P*＜0.05 was considered significant vs. sham + V. CK-MB, creatine kinase–myocardial band; DBP, diastolic blood pressure ; LVEF, left ventricular ejection fraction; LVFS, left ventricular fractional shortening; MAP, mean arterial blood pressure; SBP, systolic blood pressure.

It was recently shown that CIRP is a DAMP that is associated with sepsis. We investigated the protein levels of CIRP in heart tissues of infant rats with CLP-induced sepsis. The CIRP protein levels were higher in the CLP group than in the sham group (Figure 2A). To investigate the effect of CIRP on CLP-induced septic myocardial dysfunction, the rats were treated with C23, a CIRP inhibitor. In the CLP+C23 group, relative to the CLP infant rats, C23 treatment was associated with remarkably increased temperature, SBP, DBP, MAP, heart rate, +dP/dt_max_, −dP/dt_max_, LVEF, and LVFS (Figure 2B–K) and lower levels of CK-MB and cTnI in plasma (Figure 2L–M).

**Figure 3:**
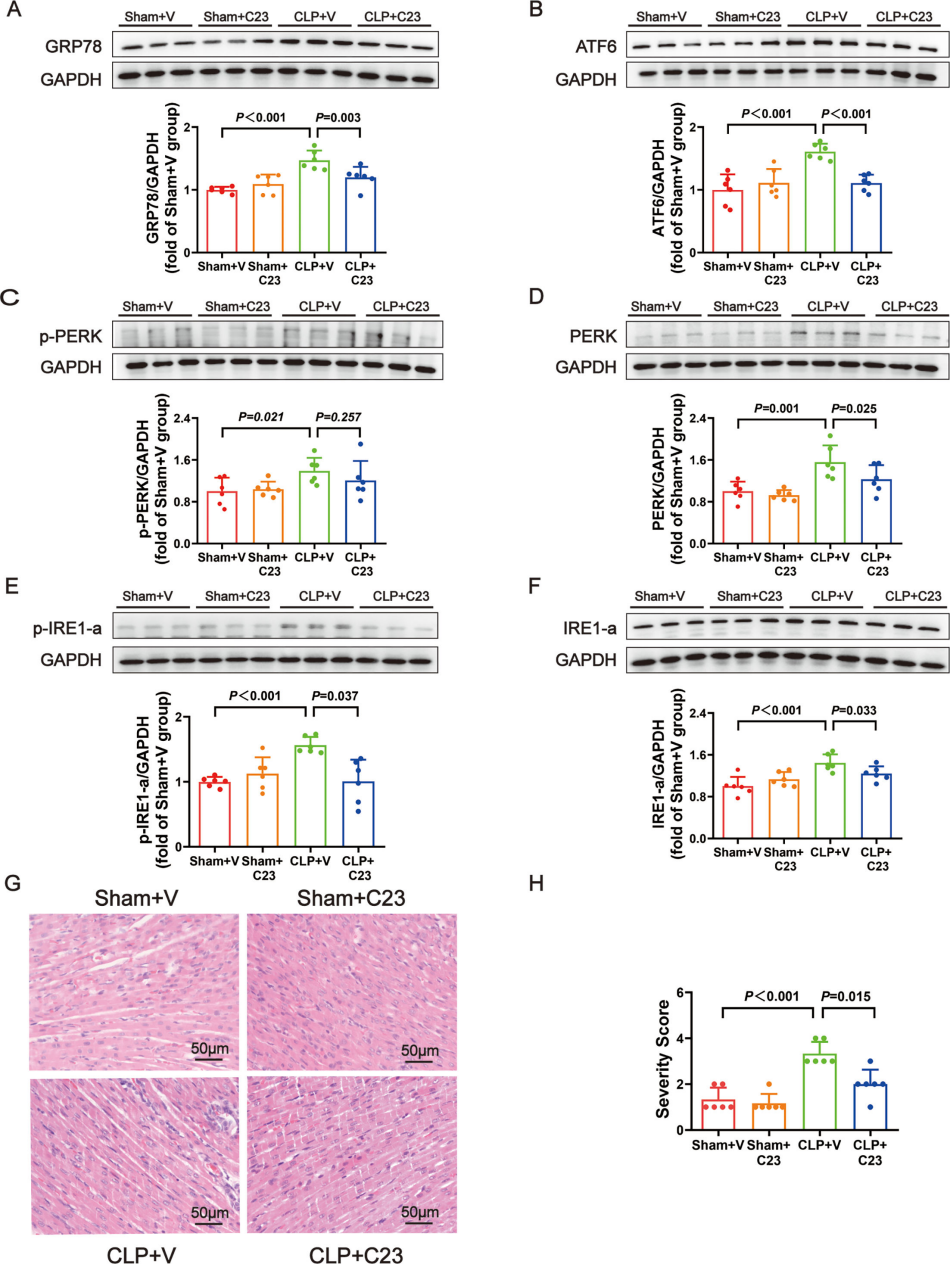
Marker proteins amount of endoplasmic reticulum stress was significantly decreased after C23 treatment. (**A–F**) Representative Western blots and quantitative analysis for GRP78, ATF6, p-PERK, PERK, p-IRE1-a, IRE1-a. GAPDH was used as the internal control. (**G**) Representative H&E staining left ventricular sections (scale bar = 50μm μm). (**H**) Severity scores of heart tissue sections. Data are mean ± SD from four independent experiments (*n* = 6). One-way analysis of variance followed by LSD test with equal variance, and the Dunnett’s T3 method was performed if there was missing variance, *P*＜0.05 was considered significant vs. sham + V. H&E, hematoxylin and eosin.

Compared with the CLP rats, the CLP+C23 rats had lower levels of the ERS markers GRP78 (Figure 3A). C23 treatment also down-regulated the protein levels of ATF6, PERK, p-IRE1-a, and IRE1-a (Figure 3B and D–F). A downward trend is observed in the level of p-PERK, although the decrease was not statistically significant (Figure 3C). In the CLP+C23 group, H&E staining showed decreased inflammatory cell infiltration, myocardial cell edema, and necrosis (Figure 3G–H). These results suggest that CIRP plays an important role during sepsis-induced cardiac dysfunction, possibly via the induction of ERS.

### ERS inhibition could reduce sepsis-induced cardiac dysfunction

**Figure 4:**
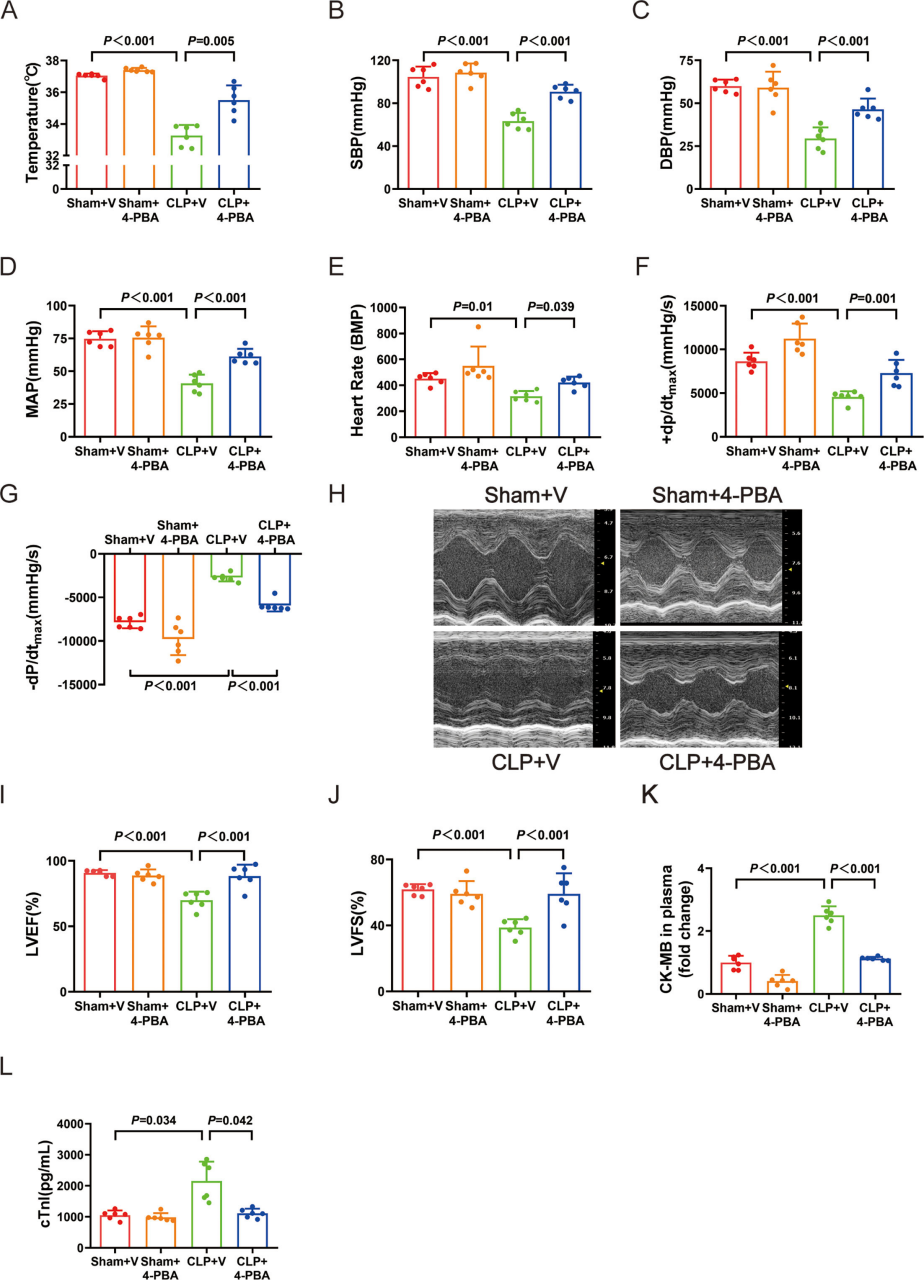
Treatment with 4-PBA (an inhibitor of endoplasmic reticulum stress) improved cardiac function in septic infant rats. (**A**) The change of temperature. (**B**) The change of SBP. (**C**) The change of DBP. (**D**) The change of MAP. (**E**) The change of heart rate. (**F**) The change of +dP/dt_max_. (**G**) The change of -−dP/dt_max_. (**H**) Representative images of M-mode by echocardiography. (**I**) The change of LVEF. (**J**) The change of LVFS. (**K**) The fold change of CK-MB in plasma. (**L**) The level of cTnI in plasma. Data are mean ± SD from four independent experiments (*n* = 6). One-way analysis of variance followed by LSD test with equal variance, and the Dunnett’s T3 method was performed if there was missing variance, *P*＜0.05 was considered significant vs. sham + V. CK-MB, creatine kinase–myocardial band; DBP, diastolic blood pressure ; LVEF, left ventricular ejection fraction; LVFS, left ventricular fractional shortening; MAP, mean arterial blood pressure; SBP, systolic blood pressure.

To further determine the role of ERS in sepsis-induced cardiac dysfunction, the effect of the ERS inhibitor 4-PBA was investigated. 4-PBA significantly increased temperature, SBP, DBP, MAP, heart rate, +dP/dt_max_, −dP/dt_max_, LVEF, and LVFS (Figure 4A–J) and down-regulated CK-MB and cTnI expression in plasma (Figure 4K–L).

**Figure 5:**
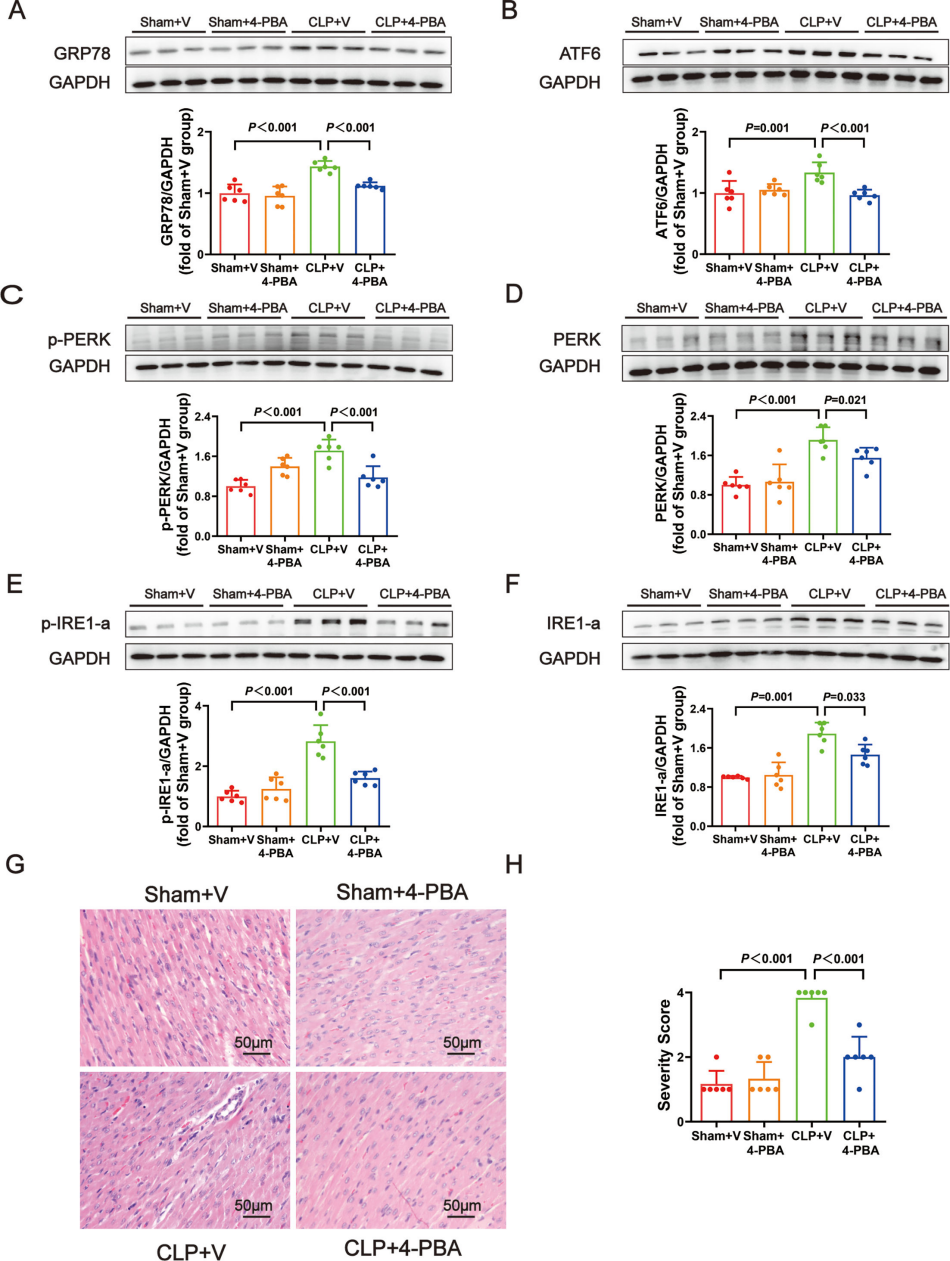
Marker proteins amount of endoplasmic reticulum stress was significantly decreased after 4-PBA treatment. (**A−F**) Representative Western blots and quantitative analysis for GRP78, ATF6, p-PERK, PERK, p-IRE1-a, IRE1-a. GAPDH was used as the internal control. (**G**) Representative H&E staining left ventricular sections (scale bar = 50μm μm). (**H**) Severity scores of heart tissue sections. Data are mean ± SD from four independent experiments (*n* = 6). One-way analysis of variance followed by LSD test with equal variance, and the Dunnett’s T3 method was performed if there was missing variance, *P*＜0.05 was considered significant vs. sham + V. H&E, hematoxylin and eosin.

Compared with the CLP rats, the CLP+4-PBA rats had lower levels of the ERS markers GRP78 (Figure 5A). 4-PBA treatment also could decrease the protein levels of ATF6, p-PERK, PERK, p-IRE1-a, and IRE1-a (Figure 5B–F). In the CLP+4-PBA group, H&E staining revealed attenuated inflammatory cell infiltration, myocardial cell edema, and necrosis (Figure 5G–H). These findings indicated that ERS inhibition could reduce sepsis-induced cardiac dysfunction.

### Supplementation of NaHS could improve cardiac function and decrease CIRP expression and ERS

H_2_S, along with NO and CO, was recently identified as a gas signaling molecule. H_2_S could play multiple roles in different biological processes. The CSE level was significantly higher in the CLP group than in the sham group (Supplementary Figure S1A ). In the CLP + PPG group, relative to the CLP group, PPG treatment further decreased temperature, SBP, DBP, MAP, heart rate, and ±dP/dt_max_ (Supplementary Figure S1C–I ) and further up-regulated CIRP expression (Supplementary Figure S1B ). H&E staining showed increased inflammatory cell infiltration, myocardial cell edema, and necrosis (Supplementary figure S1J–K) compared with that in the CLP group. These observations indicate that decreased endogenous production of H_2_S exacerbates cardiac dysfunction.

**Figure 6:**
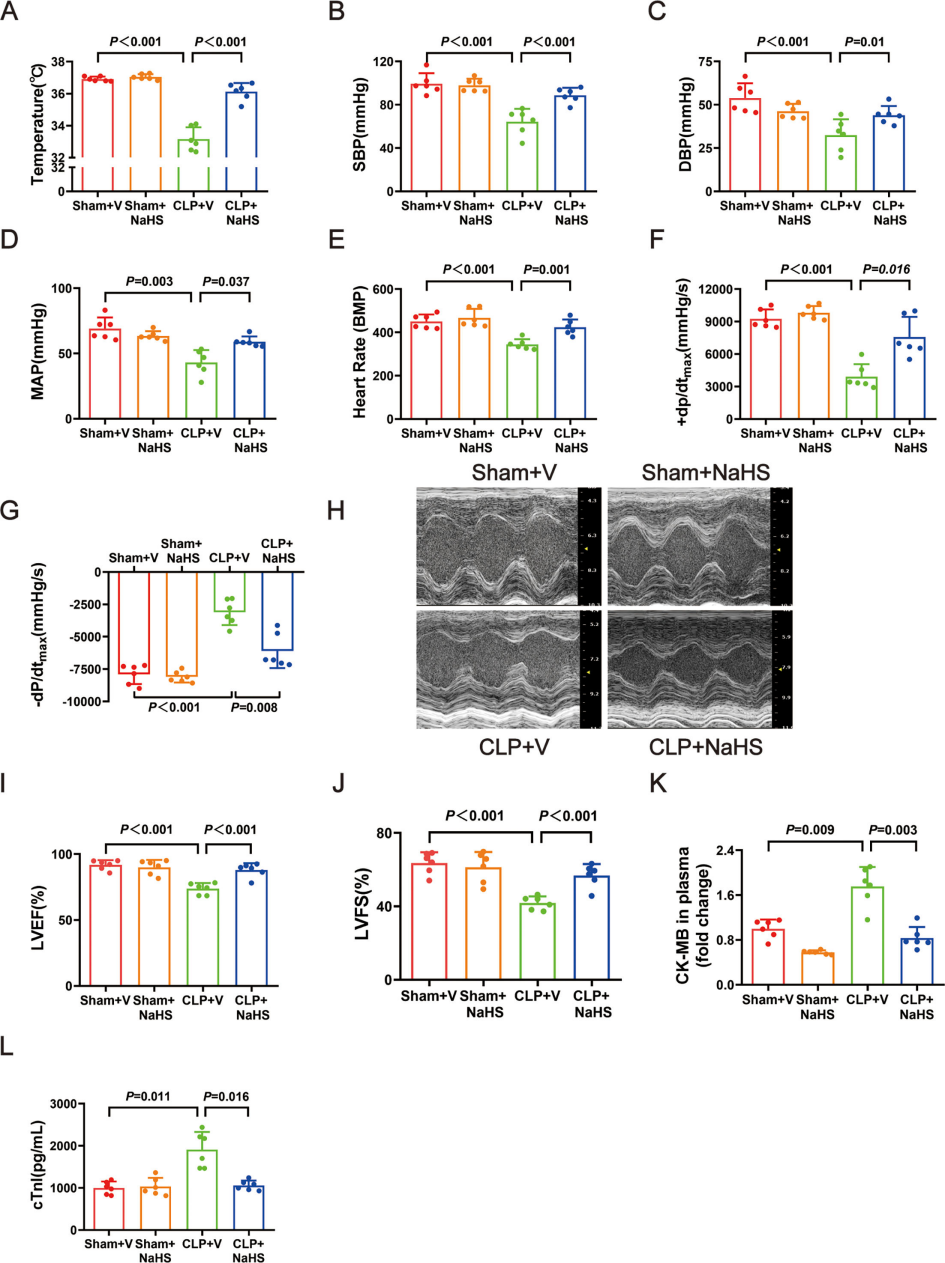
Supplying with exogenous H_2_S donor, NaHS, improved cardiac function in septic infant rats. Supplying with exogenous HS donor, NaHS, improved cardiac function in septic infant rats: (**A**) The change of temperature. (**B**) The change of SBP. (**C**) The change of DBP. (**D**) The change of MAP. (**E**) The change of heart rate. (**F**) The change of +dP/dt_max_. (**G**) The change of -−dP/dt_max_. (**H**) Representative images of M-mode by echocardiography. (**I**) The change of LVEF. (**J**) The change of LVFS. (**K**) The fold change of CK-MB in plasma. (**L**) The level of cTnI in plasma. Data are mean ± SD from four independent experiments (*n* = 6). One-way analysis of variance followed by LSD test with equal variance, and the Dunnett’s T3 method was performed if there was missing variance, *P*＜0.05 was considered significant vs. Ssham + V. CK-MB, creatine kinase–myocardial band; DBP, diastolic blood pressure ; LVEF, left ventricular ejection fraction; LVFS, left ventricular fractional shortening; MAP, mean arterial blood pressure; SBP, systolic blood pressure.

**Figure 7:**
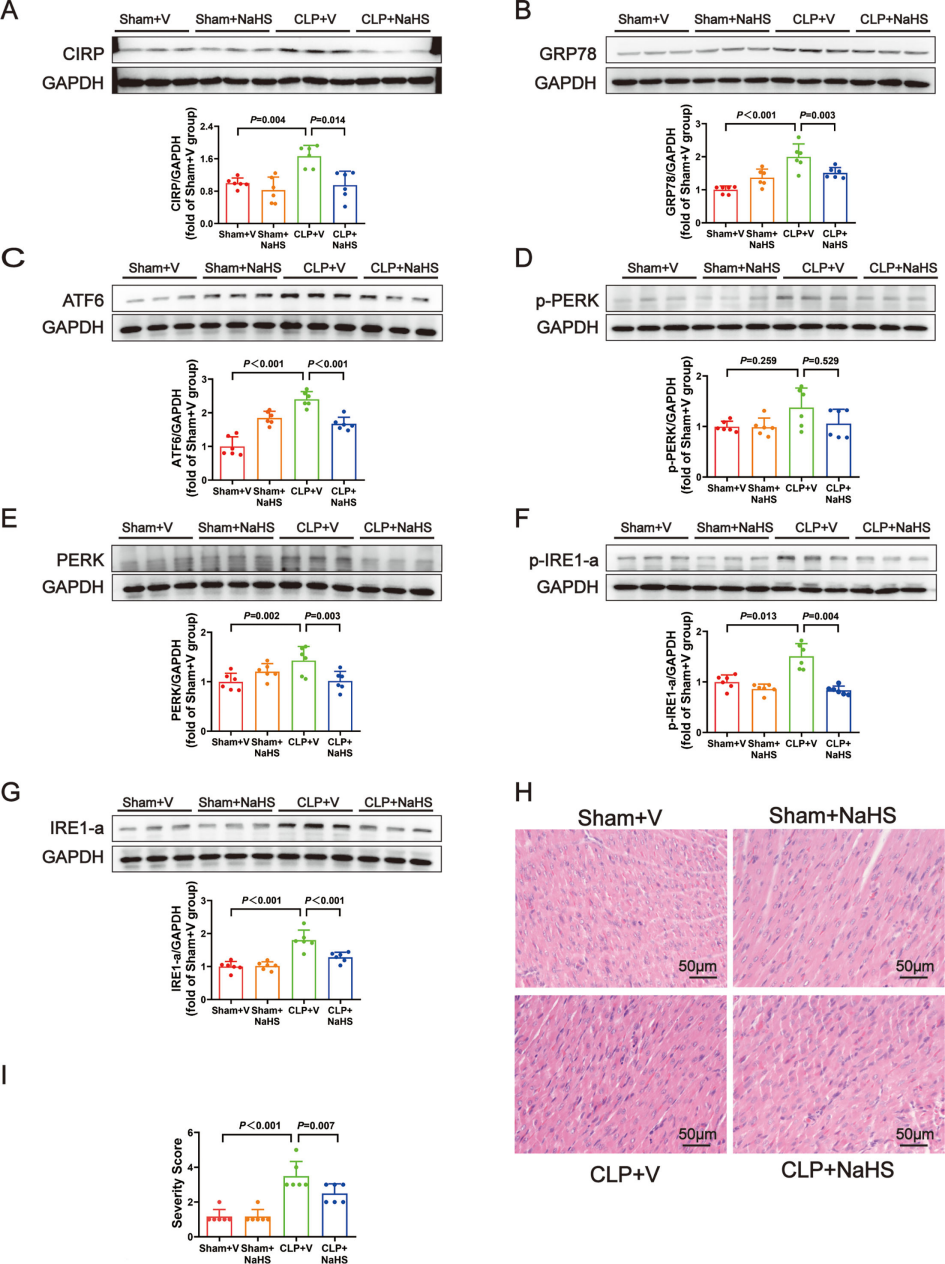
CIRP and marker proteins amount of endoplasmic reticulum stress was significantly decreased after NaHS treatment. CIRP and marker proteins amount of endoplasmic reticulum stress was significantly decreased after NaHS treatment:(**A-–G**) Representative Western blots and quantitative analysis for CIRP, GRP78, ATF6, p-PERK, PERK, p-IRE1-a, and IRE1-a. GAPDH was used as the internal control. (**H**) Representative H&E staining left ventricular sections (scale bar = 50 μm). (**I**) Severity scores of heart tissue sections. Data are mean ± SD from four independent experiments (*n* = 6). One-way analysis of variance followed by LSD test with equal variance, and the Dunnett’s T3 method was performed if there was missing variance, *P*＜0.05 was considered significant vs. Ssham + V. H&E, hematoxylin and eosin.

Therefore, supplementation of the exogenous H_2_S donor NaHS was selected for further experiments. NaHS treatment could also increase temperature, SBP, DBP, MAP, heart rate, +dP/dt_max_, −dP/dt_max_, LVEF, and LVFS (Figure 6A–J) and decrease the plasma levels of CK-MB and cTnI (Figure 6K–L). Compared with the CLP group, NaHS treatment down-regulated the protein levels of CIRP (Figure 7A) and ERS markers GRP78, ATF6, PERK, p-IRE1-a, and IRE1-a (Figure 7B–C and E–G). The level of p-PERK was also decreased, although the decrease was not statistically significant (Figure 7D). In the CLP+NaHS group, H&E staining revealed that inflammatory cell infiltration, myocardial cell edema, and necrosis were rare (Figure 7H–I). Collectively, these results indicate that H_2_S plays an important role against sepsis-induced cardiac dysfunction, possibly via the induction of CIRP and ERS.

## Discussion

As described above, in sepsis-induced cardiac dysfunction, temperature, +dP/dt_max_, and −dP/dt_max_ were lower than those in the sham group following CLP. The protein level of CIRP in cardiac tissues increased after CLP, when CIRP expression was suppressed by C23, ERS, and cardiac dysfunction was mitigated. Moreover, ERS inhibition could attenuate cardiac dysfunction. Supplementing H_2_S with a donor NaHS could reduce the CIRP level, attenuate ERS, and alleviate cardiac dysfunction. These results indicated that H_2_S exerted cardioprotective effects by inhibiting the CIRP/ERS pathway in infant rats with sepsis. The present study might provide a novel target for the treatment of sepsis in infants.

Unlike in adults, sepsis-induced cardiac dysfunction in infants was more often associated with low cardiac output, high systemic vascular resistance [23], and high mortality [24]. In the present study, the CLP group showed more cardiac dysfunction than the sham group. These results suggested that the infant rat sepsis-induced cardiac dysfunction model was successful.

Previous studies reported that organ injury was reduced in septic mice with CIRP knockout [25,26]. CIRP could act as a DAMP [14]. Although decades of research have been conducted on sepsis-associated cardiac dysfunction, the central mechanism remains unclear. Several contributory factors, including DAMPs, have been identified [27]. Evidence shown that CIRP is released into the circulation in sepsis and causes systemic inflammation [28]. In patients with sepsis, elevated concentrations of CIRP in plasma are significantly associated with poor outcomes [11]. CIRP levels in serum were increased in neonates with sepsis. In neonatal sepsis with myocardial dysfunction, CIRP interaction with TREM-1 increased inflammation, which showed that CIRP played a direct role in many of the mechanisms of sepsis-associated cardiac dysfunction in neonates [29]. It was consistent with the findings of elevated CIPR expression we observed in cardiomyocytes of CLP-induced sepsis in infant rats.

ERS is associated with various heart diseases, such as ischemic heart disease, cardiac hypertrophy, and heart failure [30]. In animal models of sepsis, the levels of ERS markers were increased in multiple organs, including the heart and liver, and the levels of these markers were directly associated with the degree of organ dysfunction, which might be the main cause of multiple-organ failure induced by sepsis [31]. Prior research has identified that excessive ERS and ATF4 activation led to the up-regulation of CIRP expression in macrophages [32]. Another study [19] showed that the levels of ERS markers in the lungs of septic mice (BiP, pIRE1α, sXBP1, CHOP, and cleavage caspase-12) almost did not increase in CIRP knockout mice compared with those in sham mice, indicating that CIRP induced ERS in septic lung injury, which was consistent with the fact that we provided C23 to neutralize CIRP and inhibit ERS. The present results show that both CIRP protein levels and ERS were decreased by C23, an inhibitor of CIRP. This suggests that C23 ameliorates ERS by inhibiting the CIRP pathway. In the present study, we demonstrated that ERS participated in sepsis-induced cardiac dysfunction in infant rats and that the levels of proteins, including GRP78, ATF6, p-PERK, PERK, p-IRE-1a, and IRE-1a were significantly increased by CLP. 4-PBA, an inhibitor of ERS, reduced cardiac dysfunction by inhibiting ERS. These observations indicated that ERS was involved in the development and progression of sepsis-induced cardiac dysfunction.

H_2_S, which is a newly identified gas signaling molecule, performs multiple biological roles in the cardiovascular system and other systems. A large number of *in vitro* and *in vivo* experiments have demonstrated that H_2_S has a protective effect in various tissues and cells [33–35]. A study showed that H_2_S could partially inhibit CHOP expression through Nrf2 activation [17]. Exogenous H_2_S reduced myocardial lipotoxicity and ERS, indicating that exogenous H_2_S inhibits ERS, lipid accumulation, and myocardial toxicity [36]. H_2_S reduced heart dysfunction caused by high-fat diet consumption by inhibiting ERS dysfunction [37]. H_2_S also reduced myocardial ischemia-reperfusion (I/R) injury by reducing excessive ERS induced by myocardial I/R [38]. Chen et al. [21] demonstrated that exogenous H_2_S inhibits inflammation and oxidative stress through TLR4/NLRP3 signaling and improved septic-induced renal dysfunction. Furthermore, Ahmad et al. [39] reported that intraperitoneal injection of H_2_S improved the survival rate of sepsis rats and reduced inflammatory reaction. As reported in our previous study, exogenous H_2_S inhibited inflammation and ERS by inhibiting the TLR4 pathway, thereby improving sepsis-induced heart dysfunction [40]. Consistent with these results, we found that H_2_S reduced the level of ERS-related proteins in the myocardia of infant rats with septic heart dysfunction and improved heart function. These results indicated that exogenous H_2_S donors regulated cardiac function by reducing ERS in infant rats with sepsis.

H_2_S could be detected in exhaled gases from septic neonates and children, which had significantly higher levels in septic neonates and children compared with controls [41]. There were few studies on the regulation of CIRP by H_2_S. A study has shown that H_2_S reduced HG-induced myocardial injury and inflammation by up-regulating CIRP expression in H9c2 cardiomyocytes [42]. Another study suggested that in mice with heart failure, down-regulation of CIRP expression predisposed heart cells to apoptosis [43]. In the present study, we obtained contrasting results. H_2_S could down-regulate CIRP expression to reduce heart dysfunction and inflammation in infant rats with sepsis, which supported that H_2_S was an important mechanism regulating CIRP in sepsis.

The present study had some limitations. First, we did not use knockout animals or agonists of CIRP/ERS to validate the relationship between CIRP and ERS in infant rats with septic cardiac dysfunction. Second, the present study did not provide direct evidence of the effect of H_2_S on CIRP/ERS, and H₂S might mitigate the inflammatory response through alternative pathways, thereby CIRP/ERS. Third, due to the absence of suitable cellular models for simulating myocardial damage resulting from sepsis in pediatric populations, no further studies had been undertaken at the cellular level. However, we used inhibitors of CIRP and ERS to provide initial exploratory results, which might be the direction of our further study.

## Conclusions

In the present study, using a septic infant rat model, we show that H_2_S significantly alleviates sepsis-induced cardiac dysfunction, probably by inhibiting CIRP and ERS. The present study provides new strategies and a new target for the prevention and treatment of infant sepsis.

## Supplementary material

Online supplementary figure 1

## Data Availability

The data that support the findings of this study are available from the corresponding author upon reasonable request.

## Abbreviations

CIRP: cold-inducible RNA-binding protein
CK-MB: creatine kinase–myocardial band
CLP: cecal ligation and puncture
DAMPs: damage-associated molecular patterns
DBP: diastolic blood pressure
ECL: enhanced chemiluminescence
ERS: endoplasmic reticulum stress
H&E: hematoxylin and eosin
LSD: Least-Significant Difference
LVEF: left ventricular ejection fraction
LVFS: left ventricular fractional shortening
MAP: mean arterial blood pressure
PAMPs: pathogen-associated molecular patterns
SBP: systolic blood pressure
TBST: Tris-buffered saline with Tween solution
