# Supplementary material for: Hydrogen sulfide attenuates sepsis-induced cardiac dysfunction in infant rats by inhibiting the expression of cold-inducible RNA-binding protein
Source: Biosci Rep. 2025 Feb 17;45(2):BSR20241398. doi: 10.1042/BSR20241398 (PMC12096945; doi:10.1042/BSR20241398)

Supplementary Figure S1

**Supplementary Fig. 1.** **Treatment with propargylglycine (PPG), an inhibitor of endogenous generating, further damage cardiac function in septic infant rats: (A-B)** Representative Western blots and quantitative analysis for CSE and CIRP. GAPDH was used as the internal control. **(C)** The change of temperature. **(D)** The change of SBP. **(E)** The change of DBP. **(F)** The change of MAP. **(G)** The change of heart rate. **(H)** The change of +dP/dt_max_. **(I)** The change of -dP/dt_max_. **(J)** Representative H&E staining left ventricular sections (scale bar =50μm). **(K)** Severity scores of heart tissue sections. Data are mean ± SD from two independent experiments (n = 6). Independent t-tests, *P*＜0.05 was considered significant vs. Sham. Data are mean ± SD from four independent experiments (n = 6). One-way analysis of variance followed by LSD test with equal variance, and the Dunnett’s T3 method was performed if there was missing variance, *P*＜0.05 was considered significant vs. Sham+V.


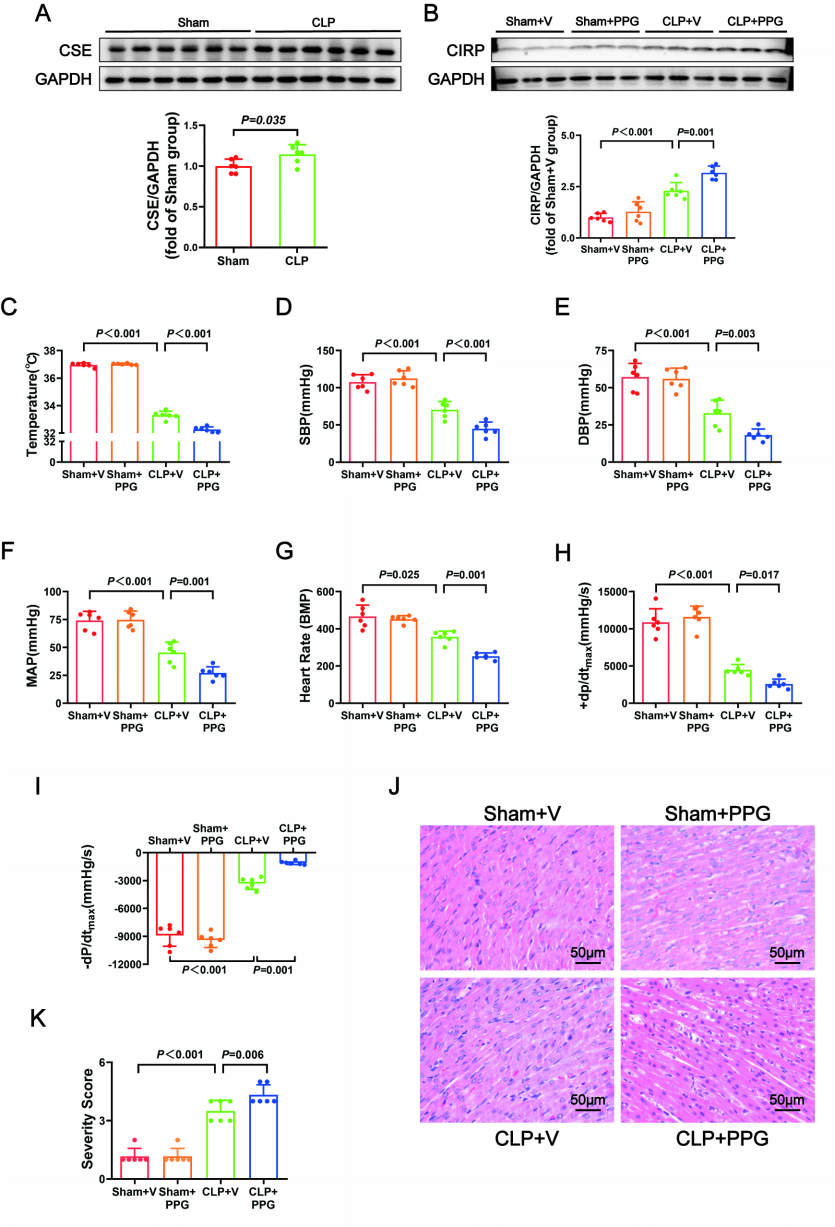

Supplement: Online supplementary figure 1 [file bsr-45-02-bsr-2024-1398-s001.docx]
